# Prediction of a competing endogenous RNA co‐expression network as a prognostic marker in glioblastoma

**DOI:** 10.1111/jcmm.15957

**Published:** 2020-10-13

**Authors:** Qunlong Peng, Runmin Li, Ying Li, Xiaoqian Xu, Wensi Ni, Huiran Lin, Liang Ning

**Affiliations:** ^1^ College of Pharmacy Xiangnan University Chenzhou China; ^2^ College of Traditional Chinese Medicine Shandong University of Traditional Chinese Medicine Jinan China; ^3^ School of Nursing Nanchang University Nanchang China; ^4^ Department of Gynaecology and Obstetrics Shenzhen University General Hospital Shenzhen China; ^5^ Department of Pediatrics Shenzhen University General Hospital Shenzhen China; ^6^ Laboratory Animal Management Office Public Technology Service Platform Shenzhen Institutes of Advanced Technology Chinese Academy of Sciences Shenzhen China; ^7^ Guangdong Key Laboratory for Biomedical Measurements and Ultrasound Imaging School of Biomedical Engineering Shenzhen University Health Science Center Shenzhen China; ^8^ Key Laboratory of Optoelectronic Devices and Systems College of Physics and Optoelectronic Engineering Shenzhen University Shenzhen China

**Keywords:** co‐expression network, competing endogenous RNA, glioblastoma, prediction, prognostic marker

## Abstract

Due to its high proliferation capacity and rapid intracranial spread, glioblastoma (GBM) has become one of the least curable malignant cancers. Recently, the competing endogenous RNAs (ceRNAs) hypothesis has become a focus in the researches of molecular biological mechanisms of cancer occurrence and progression. However, there is a lack of correlation studies on GBM, as well as a lack of comprehensive analyses of GBM molecular mechanisms based on high‐throughput sequencing and large‐scale sample sizes. We obtained RNA‐seq data from The Cancer Genome Atlas (TCGA) and Genotype‐Tissue Expression (GTEx) databases. Further, differentially expressed mRNAs were identified from normal brain tissue and GBM tissue. The similarities between the mRNA modules with clinical traits were subjected to weighted correlation network analysis (WGCNA). With the mRNAs from clinical‐related modules, a survival model was constructed by univariate and multivariate Cox proportional hazard regression analyses. Thereafter, we carried out Gene Ontology (GO) and the Kyoto Encyclopedia of Genes and Genomes (KEGG) enrichment analyses. Finally, we predicted interactions between lncRNAs, miRNAs and mRNAs by TargetScan, miRDB, miRTarBase and starBase. We identified 2 lncRNAs (NORAD, XIST), 5 miRNAs (hsa‐miR‐3613, hsa‐miR‐371, hsa‐miR‐373, hsa‐miR‐32, hsa‐miR‐92) and 2 mRNAs (LYZ, PIK3AP1) for the construction of a ceRNA network, which might act as a prognostic biomarker of GBM. Combined with previous studies and our enrichment analysis results, we hypothesized that this ceRNA network affects immune activities and tumour microenvironment variations. Our research provides novel aspects to study GBM development and treatment.

## 1 INTRODUCTION

Glioblastoma (GBM) ranks the most prevalent malignant cancer of the central nervous system and has a mortality rate of approximately 3.19 per 100 000.^1^ Intensive therapy is considered to be the standard treatment of GBM.^2^ However, to date, although the standard treatment has been applied, its high proliferative capacity and fast intracranial dissemination make it the least curable cancer, with a median overall survival of approximately 15 months.^3^ In recent years, vast quantities of valuable information have accumulated by expanding big data analyses in molecular biology and developing targeted therapy techniques, which has laid a solid foundation for cancer research.^4^ However, the detailed molecular mechanisms of GBM still remain unclear, which brings difficulties to its diagnosis and treatment. Thus, finding novel molecular mechanisms and biomarkers of GBM to enable the early diagnosis and treatment precision has become a hotspot in GBM research.

Currently, studies on competing endogenous RNA (ceRNA) co‐expression networks are providing new perspectives on cancer pathogenesis at the molecular level. Acting as the key link in ceRNA co‐expression networks, lncRNAs regulate gene expression through competitive binding with specific miRNAs, sequestering RNA‐binding proteins and influencing nuclear transcription.^5^, ^6^ Through their roles in ceRNA co‐expression networks, lncRNAs significantly affect the biological processes of brain cancer. For example, Zhang found GBM cells remodel the tumour microenvironment to promote tumour chemotherapy‐resistance by secreting oncogenic lncSBF2‐AS1‐enriched exosomes.^7^ LncRNA AC016405.3 was found to suppress proliferation and metastasis through modulating TET2 by sponging of miR‐19a‐5p in GBM cells.^8^ The sponging of miR‐885‐3p by lncRNA HOXB‐AS1 could further affect the expression of HOXB2, and this process regulates the proliferation and migration of GBM.^9^ Other evidence showed that LINC01579 could competitively bind with miR‐139‐5p to regulate EIF4G2 and thus lead to cell proliferation and apoptosis in GBM.^10^ Therefore, these studies have shown that the lncRNA‐miRNA‐mRNA ceRNA co‐expression network is implicated in the development of GBM. However, publications on GBM are limited, and a comprehensive analysis of GBM molecular mechanisms based on high‐throughput sequencing and on a large‐scale sample size is lack.

In the present study, we extracted RNA‐seq data from Genotype‐Tissue Expression (GTEx) and The Cancer Genome Atlas (TCGA). Furthermore, differentially expressed mRNAs were identified and applied to weighted correlation network analysis (WGCNA) for screening modules related to clinical traits. Among these modules, we set up a survival model by Cox proportional hazard regression analysis to predict GBM outcomes. Finally, combining information from multiple databases, we predicted key lncRNAs and miRNAs to weave a ceRNA network for explaining the molecular mechanism of GBM.

## 2 MATERIALS AND METHODS

### 2.1 Data processing and differential expression analysis

The RNA sequence data of 5 normal brain tissues and 168 GBM tissues were obtained from TCGA database (https://portal.gdc.cancer.gov/). Clinical data such as patient age, gender, overall survival time and overall survival state were also downloaded from TCGA. Expression data of 105 normal brain tissues were obtained from the GTEx (https://gtexportal.org/home/datasets) database. Complete descriptions of the donor's age, gender, biospecimen procurement methods and sample fixation were presented in the GTEx official annotation. With the assistance of the R package (limma), differently expressed mRNAs were retrieved according to *P* < .05 and absolute log2‐fold change (FC) > 2.^11^ For excluding non‐coding mRNA interference, Ensembl ID was used to identify and obtain protein‐coding mRNA information for further study. To visualize the differentially expressed mRNAs, volcano plots were generated using the ggplot2 package for the R platform.

### 2.2 GO and KEGG pathway enrichment analyses of differentially expressed mRNAs

We performed the Kyoto Encyclopedia of Genes and Genomes (KEGG) pathway enrichment analyses and Gene Ontology (GO) with the R package (clusterProfiler).^12^, ^13^, ^14^ The biological processes, cell components, molecular function and KEGG pathways of the differentially expressed mRNAs were retrieved with a cut‐off criterion of *P* < .05 and visualized by the R packages ggplot2 and GOplot.

### 2.3 Weighted correlation network analysis

We integrated the data from the differentially expressed mRNAs to perform weighted correlation network analysis (WGCNA). The R package ‘WGCNA’ was adopted to detect traits‐related modules.^15^ Herein, we set the soft‐thresholding power as 6 and scale‐free R2 as >0.85 to figure out key modules. The modules were then applied to analyse their relationship with GBM clinical traits using Pearson's correlation test, and adjusted *P* < .05 was considered significant. GO enrichment analysis was applied in order to explain the biological role of the modules.

### 2.4 Cox proportional hazard regression analysis

First, we conducted a univariate Cox proportional hazard regression analysis to determine the relationship of the expression levels of mRNAs from modules related to clinical traits with overall survival (OS). Second, multivariate Cox proportional hazard regression analysis was carried out for setting up a survival model. Finally, survival analysis and receiver operating characteristic (ROC) analyses of the model were performed. The forest plots, risk score plot, heat map, area under the receiver operating characteristic curve (AUC) and survival curve of the survival model were visualized.

### 2.5 Construction of ceRNA co‐expression network

Combining the above research results, we predicted targeting miRNAs of the mRNAs with high prognosis values by using the online bioinformatics tools TargetScan (https://www.targetscan.org/), miRDB (https://www.mirdb.org/miRDB/) and miRTarBase (https://mirtarbase.mbc.nctu.edu.tw/). Different algorithms and prediction models are used by these databases for predicting miRNAs. TargetScan operates by searching for conserved sites and ranks the results by predicted targeting efficacy.^16^, ^17^ By analysing thousands of miRNA‐target interactions (MTIs) from high‐throughput sequencing experiments, miRDB can predict biologically relevant interactions between miRNAs and genes.^18^ The miRTarBase contains more than three hundred and sixty thousand MTIs that have been validated experimentally by reporter assays, Western blots, microarrays and next‐generation sequencing experiments.^19^ We merged these predicted miRNAs to improve the reliability of the results. Millions of RNA‐binding sites from 108 CLIP‐seq data sets have been analysed and deposited in starBase (https://starbase.sysu.edu.cn/), which provides tools to predict relevant lncRNA‐miRNA interactions.^20^ Ultimately, a ceRNA co‐expression network containing lncRNAs, miRNAs and mRNAs was constructed and visualized by Cytoscape software.

## 3 RESULTS

### 3.1 Identification of differentially expressed mRNAs in GBM

**FIGURE 1:**
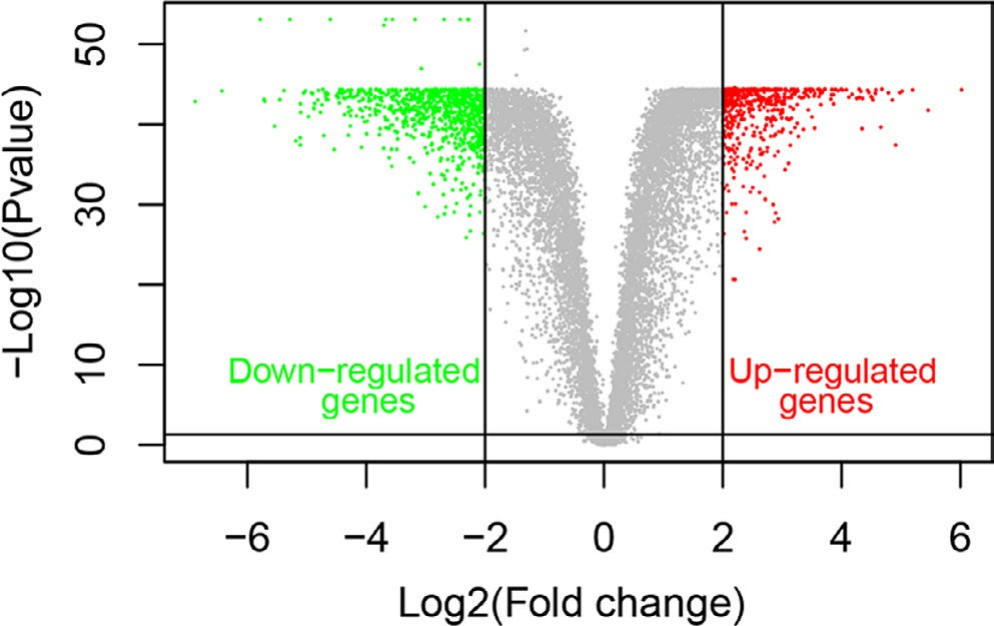
Volcano plot of differentially expressed mRNAs. Red spots represent up‐regulated mRNAs, and green spots represent down‐regulated mRNAs

The general condition and clinical characteristics of the GBM patients are presented in Table 1. The expression of mRNAs level in 110 normal brain tissue samples and 168 GBM cases was explored for further study. When *P* < .05 and |log2 FC|>2 were used as cut‐off criteria, 1347 differentially expressed mRNAs were standardized and identified via the limma R package, which included 516 up‐regulated and 831 down‐regulated mRNAs. A volcano plot was applied to illustrate the down‐regulated and up‐regulated mRNAs (Figure 1).

**Table 1:** The clinical characteristics of GBM patients

	Alive (n = 30)	Dead (n = 137)	Total (n = 167)
Gender			
Female	12 (40%)	47 (34%)	59 (35%)
Male	18 (60%)	90 (66%)	108 (65%)
Age			
Mean (SD)	54.37 (16.41)	60.30 (12.68)	59.23 (13.56)
Median [MIN, MAX]	54 [21, 82]	62 [21, 89]	60 [21, 89]
Overall survival time			
Mean (SD)	334.27 (261.24)	447.42 (404.76)	427.10 (384.76)
Median [MIN, MAX]	220 [13, 958]	382 [5, 2681]	360 [5, 2681]

### 3.2 GO, KEGG pathway enrichment analyses

**FIGURE 2:**
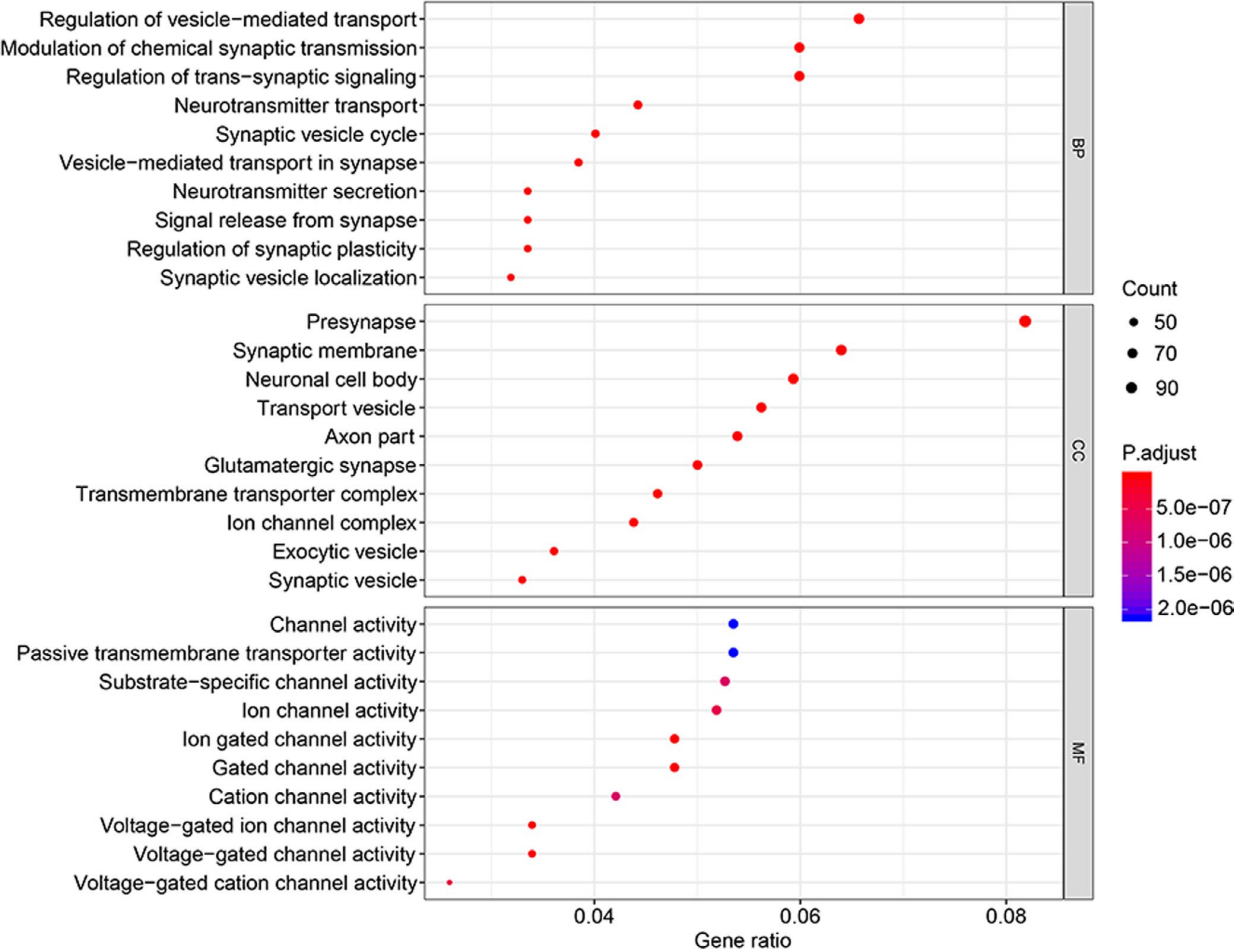
GO enrichment analyses of differentially expressed mRNAs

**FIGURE 3:**
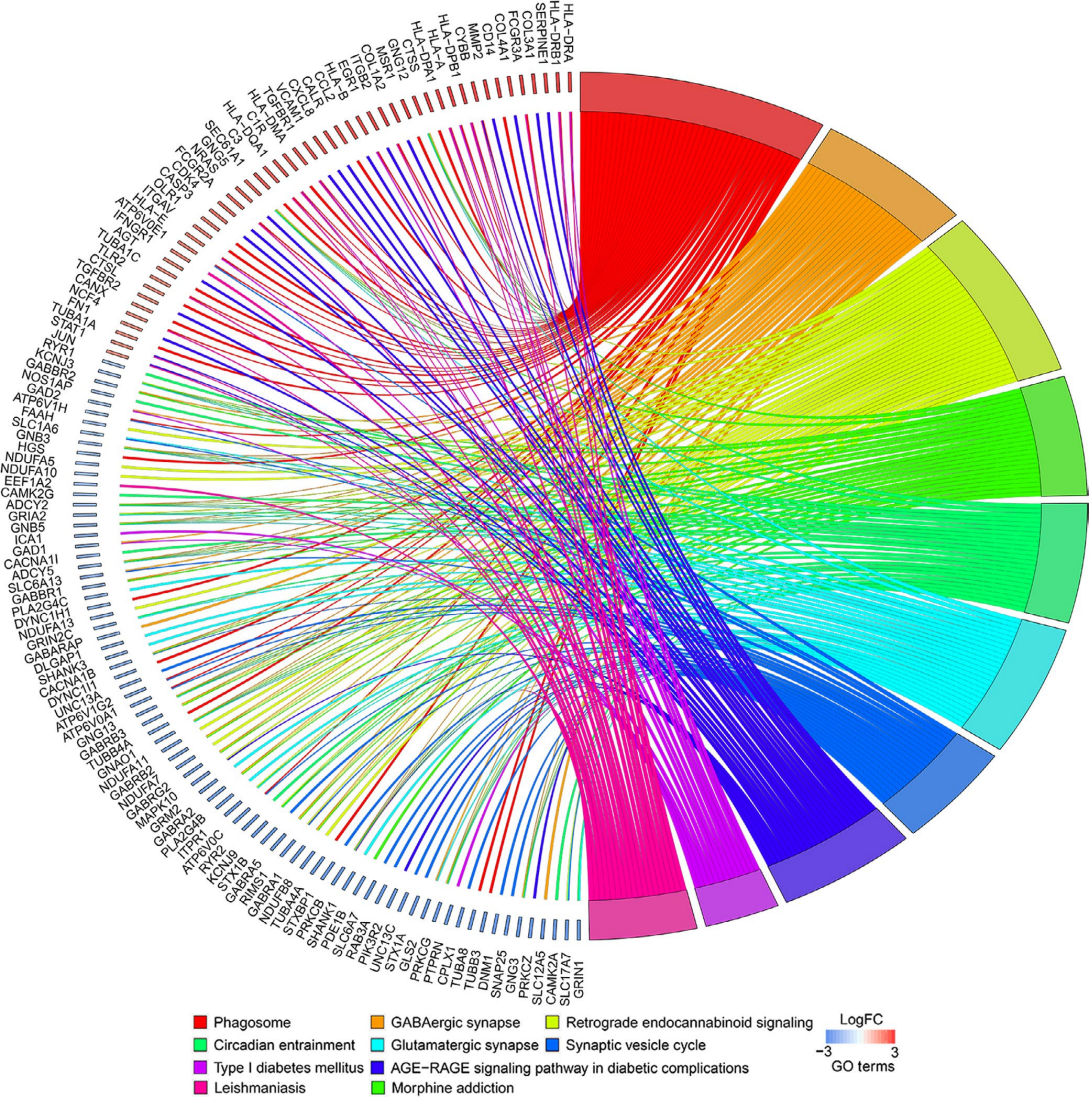
KEGG pathway enrichment analyses of differentially expressed mRNAs

To explore the biological characteristics of the differentially expressed mRNAs, GO enrichment analyses were performed with a cut‐off criterion of *P* < .05. As shown in Figure 2, in the ‘biological processes’ group, differentially expressed mRNAs were mainly enriched in synapse and vesicle‐mediated transport, such as ‘regulation of vesicle‐mediated transport’, ‘synaptic vesicle cycle’ and ‘modulation of chemical synaptic transmission’. Moreover, in the ‘cell component’ group, differentially expressed mRNAs were identified to related to transmembrane transport‐related structures, for instance ‘synaptic membrane’, ‘transport vesicle’, ‘transmembrane transporter complex’ and ‘synaptic vesicle’. In addition, ‘molecular function’ analysis verified that these differentially expressed mRNAs were correlated with all kinds of channel activity: ‘substrate‐specific channel activity’, ‘voltage‐gated ion channel activity’ and ‘voltage‐gated cation channel activity’. KEGG pathway enrichment analysis using *P* < .05 as a cut‐off criterion demonstrated that the differentially expressed mRNAs were related to the complex biological behaviour of GBM such as ‘retrograde endocannabinoid signalling’, ‘phagosome’, ‘GABAergic synapse’, ‘synaptic vesicle cycle’ and ‘glutamatergic synapse’ (Figure 3). These most significantly enriched GO terms and KEGG pathways indicated the interactions of differentially expressed mRNAs at the functional level.

### 3.3 WGCNA analysis applied to differentially expressed mRNAs

**FIGURE 4:**
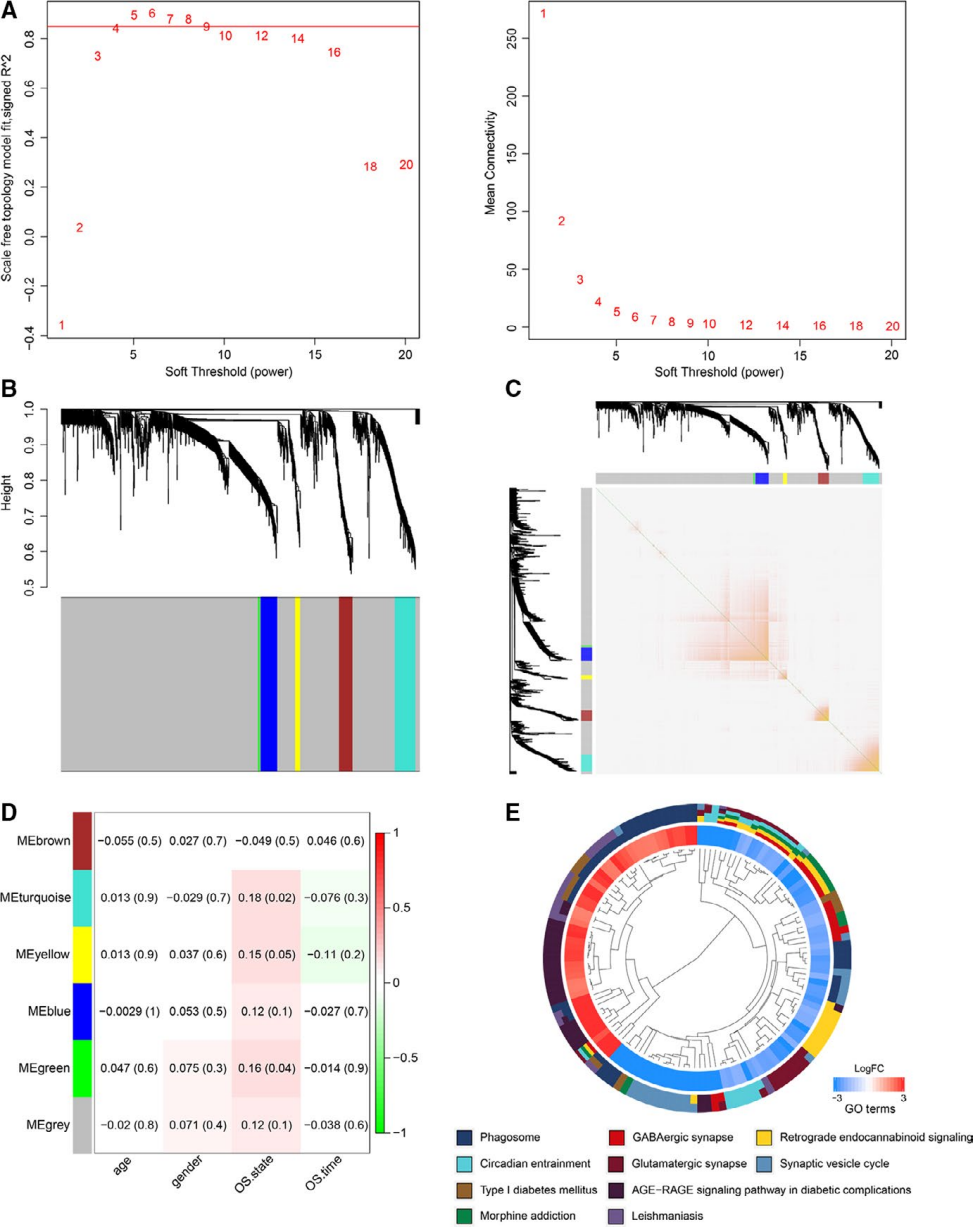
WGCNA identified critical modules correlating with GBM clinical traits. A, Analysis of the scale‐free fit index for various soft‐threshold powers and mean connectivity for various soft‐threshold powers. B, Cluster dendrogram of the co‐expression network modules was produced based on topological overlaps in the mRNAs. C, Heat map of topological overlap matrix in the mRNA modules is shown. D, Correlation between modules and traits. The number on the left in each cell refers to the correlation efficient of each module in the trait, and the number on the right is the corresponding *P*‐value. E, Clustering plot describing the top 10 results of the GO enrichment analysis

In the present work, mRNA modules among the 1347 differentially expressed mRNAs were analysed using the WGCNA R package. As shown in Figure 4A, softpower 6 and scale‐free R2 as > 0.85 were chosen as the thresholds to identify co‐expressed mRNA modules. Five mRNA colour modules were identified and the heat maps of topological overlap matrix (TOM) are presented in Figure 4B and Figure 4C. Then, mRNAs in the 5 different coloured modules were continuously used to analyse their correlation with GBM clinical traits using Pearson's correlation test and *P* < .05 was considered significant. The green module and turquoise module, which included 83 mRNAs, displayed strong relationships with the overall survival state of the GBM cases (Figure 4D). These 83 mRNAs were further subjected to GO enrichment analyses for explaining their biological roles. As shown in Figure 4E, the enrichment analysis revealed that the mRNAs from the modules were most related to ‘phagocytosis’, ‘neutrophil mediated immunity’ and ‘immune response‐regulating cell surface receptor signalling pathway’.

### 3.4 Identification of prognosis‐related genes

**FIGURE 5:**
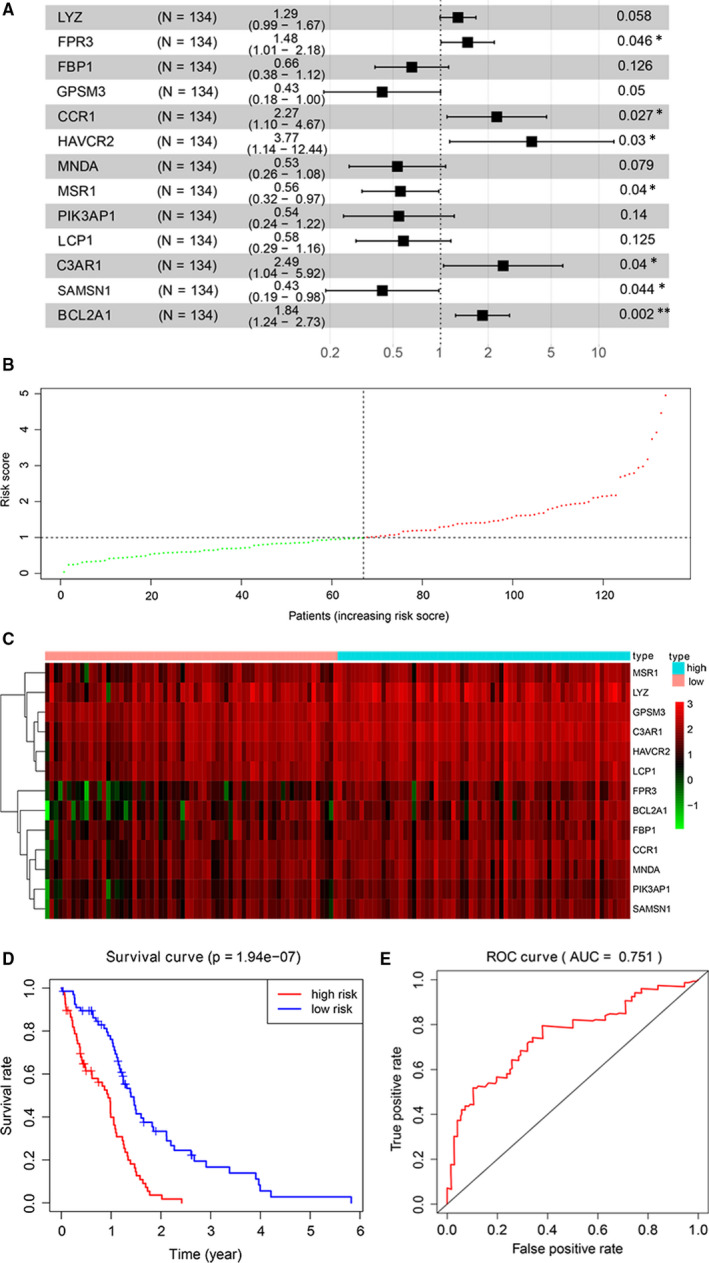
The establishment of a prognostic assessment model by Cox proportional hazard regression analysis. A, Related mRNA parameters in the prognostic assessment model calculated by multivariate Cox proportional hazard regression analysis. B, The GBM samples were divided into low‐ and high‐risk groups based on the multivariate Cox score. C, An expression heat map of the 13 mRNAs in the high‐risk or low‐risk groups is shown. D, Receiver operating characteristic analysis of the 13‐mRNA model was performed. E, Kaplan‐Meier survival analysis of the 13‐mRNAs model was performed

Next, we randomly selected 80% of all of the GBM samples (n = 134) from TCGA database as the test group. A univariate Cox proportional hazard regression analysis was applied to determine the relationship of the expression levels of 83 mRNAs with overall survival (OS). A total of 28 mRNAs were obtained by the threshold of *P*‐value < .05 (Table 2). The abovementioned 28 mRNAs were subjected to a multivariate Cox proportional hazard regression analysis. We then set up a survival model for OS with 13 mRNAs as follows: LYZ + FPR3 + FBP1 + GPSM3 + CCR1 + HAVCR2 + MNDA + MSR1 + PIK3AP1 + LCP1 + C‐3AR1 + SAMSN1 + BCL2A1 (Figure 5A). The GBM samples were divided into predicted low‐ (n = 67) and high‐risk groups (n = 67) according to the multivariate Cox score result as shown in Figure 5B. Moreover, the expression heat map of the 13 mRNAs in the high‐risk or low‐risk groups is shown in Figure 5C. We further estimated the accuracy of the 13‐mRNA signature for predicting survival. Kaplan‐Meier survival curves depicted that, compared with low‐risk patients, those predicted high risk had significantly shorter OS (*P* = 1.94e − 07, Figure 5D). Receiver operating characteristic (ROC) analysis to compare the sensitivity and specificity of the survival prediction of our models was subsequently carried out. TCGA data set indicated that AUC of the 13‐mRNAs signature was 0.751, showing high sensitivity and specificity for prognostication (Figure 5E).

**Table 2:** The results of univariate Cox proportional hazard regression analysis

ID	HR	*P*‐value
LYZ	1.187	.013
SPI1	1.286	.032
FPR3	1.256	.008
TYMP	1.244	.030
FBP1	1.234	.047
FERMT3	1.397	.008
APOC1	1.251	.021
CTSS	1.236	.023
FCGR2A	1.203	.049
GPSM3	1.290	.047
CCR1	1.322	.007
MAFB	1.199	.044
PLEK	1.334	.011
HAVCR2	1.311	.015
MNDA	1.239	.035
MSR1	1.206	.042
PIK3AP1	1.268	.039
UCP2	1.347	.014
NPC2	1.278	.032
C1QA	1.226	.039
LCP1	1.256	.042
PTAFR	1.282	.037
SLC7A7	1.282	.041
C3AR1	1.291	.017
ABI3	1.338	.031
SAMSN1	1.279	.035
LAPTM5	1.268	.045
BCL2A1	1.240	.006

### 3.5 Construction of the ceRNA co‐expression network

**FIGURE 6:**
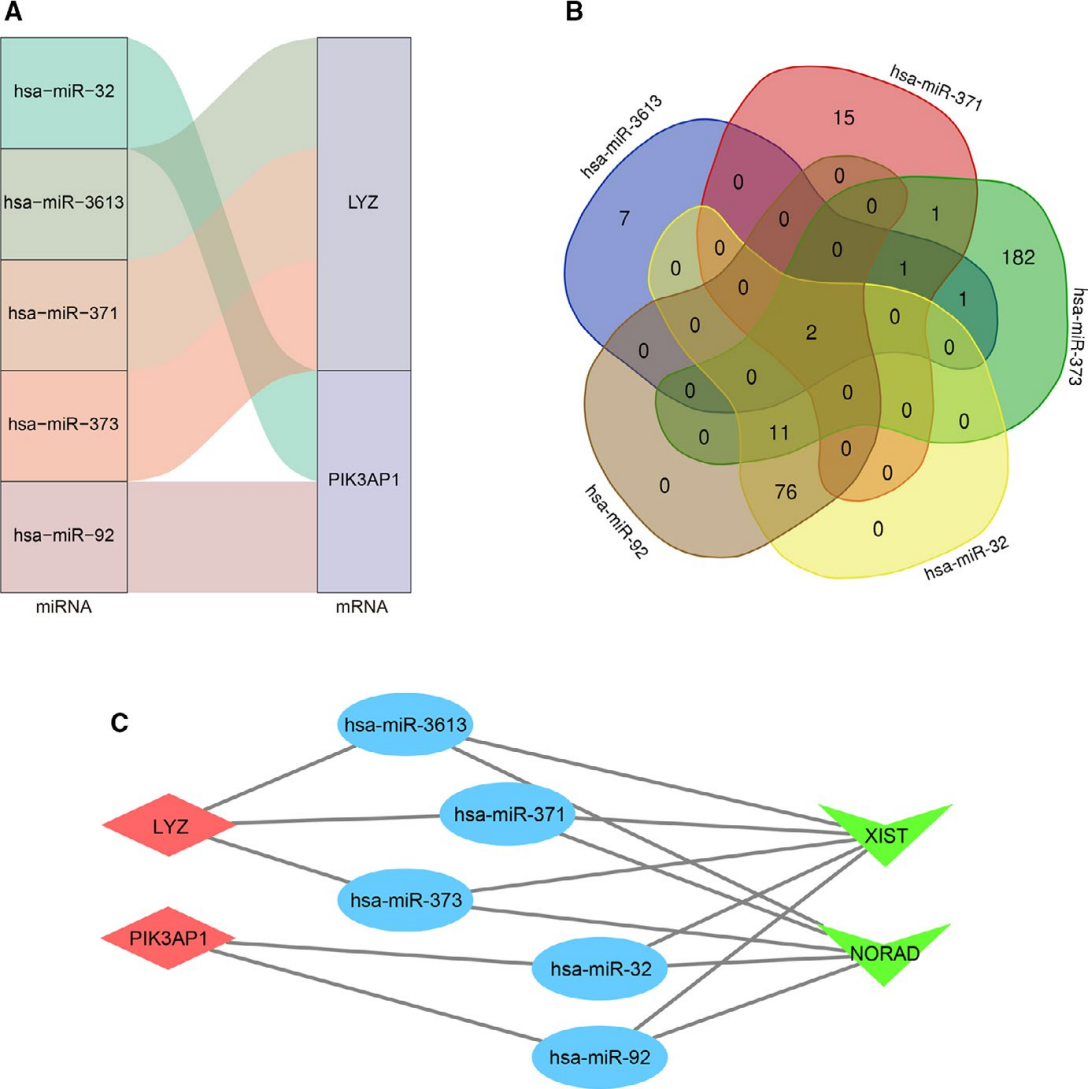
Construction of the ceRNA co‐expression network. A, The relationship between the mRNAs and their corresponding miRNAs is shown. B, Overlapping lncRNAs were analysed by the predicted lncRNAs of 5 miRNAs. C, The lncRNA‐miRNA‐mRNA ceRNA co‐expression network was constructed via 2 lncRNAs, 5 miRNAs and 2 mRNAs for GBM prognosis

Combining the above research results, we predicted targeting miRNAs of the 13 mRNAs in the survival model by TargetScan, miRDB and miRTarBase. Furthermore, mRNAs without predicted miRNA intersections in the three databases were discarded (Figure 6A). Targeted lncRNAs of these predicted miRNAs were screened by starBase and overlapped for seeking co‐regulatory pathways (Figure 6B). We merged these predicted results, found LYZ was related to hsa‐miR‐3613, hsa‐miR‐371, hsa‐miR‐373 and hsa‐miR‐32 and found that hsa‐miR‐92 regulated PIK3AP1. Moreover, lncRNAs XIST and NORAD were targeted to all of the 5 predicted miRNAs. Ultimately, a ceRNA co‐expression network containing lncRNAs, miRNAs and mRNAs was constructed and visualized by Cytoscape software (Figure 6C).

## 4 DISCUSSION

Glioblastoma is considered as an incurable disease because of its high proliferative capacity and active infiltrative growth.^21^ Finding possible molecular mechanisms and potential biomarkers of GBM is the urgent task at hand.^22^ With the development of human genome research, the importance of non‐coding genes’ regulatory effects is becoming increasingly clear. LncRNAs acts as a critical role in the occurrence, growth, metastasis, prognosis and treatment of cancers.^23^, ^24^ The ceRNA hypothesis describes regulatory networks among protein‐coding mRNAs and non‐coding RNAs, bridges the gap between lncRNA unconventional expression and mRNA expression regulation and lays a foundation for studying the mechanisms of specific lncRNAs.^5^

The rapid development of bioinformatics methods provides methodological support for exploring high‐throughput sequencing data. We applied WGCNA and Cox proportional hazard regression analysis for processing and analysing GBM sequencing and clinical data. It is estimated that on average each mRNA interacts with four to eight other mRNAs and is involved in 10 biological functions.^25^, ^26^ Based on that, WGCNA established the incorporating modules and exploring clinical trait relevance as its core algorithms. Through soft‐threshold filtering, co‐expression matrix constructing, weighted network establishing and hierarchical clustering, module‐trait networks can be constructed for understanding the clinical relevance of biomarkers accurately.^27^ Cox proportional hazard regression models have the function of processing truncated survival time, simultaneously analysing various variables, and has no requirement on the distribution type of the survival function of data.^28^ All of these advantages make it the most common modelling tool in survival analysis. In the evaluation of cancer prognosis, univariate and multivariate Cox proportional hazard regression analysis has been widely used to screen prognostic related mRNAs and construct survival models.

Herein, based on the RNA‐seq data and clinical data from GBM patients, 1347 differentially expressed mRNAs were screened out and incorporated into WGCNA to obtain prognostic related modules. To demonstrate the biological functions of the differentially expressed mRNAs and mRNA modules, enrichment analysis was conducted. In addition, we established a survival model though univariate regression analysis and multivariate regression analysis of mRNAs derived from the clinically relevant modules. A ceRNA co‐expression network for prognostication was finally constructed by querying online databases and overlapping the prediction results. Ultimately, 2 lncRNAs, 5 miRNAs and 2 mRNA were identified as prognosis biomarkers.

The GO and KEGG enrichment analyses suggested that the differentially expressed mRNAs and clinically related modules were extensively associated with ‘regulation of vesicle‐mediated transport’, ‘synaptic vesicle’, ‘voltage‐gated ion channel activity’ and ‘neutrophil degranulation’. Existing evidence shows these processes and structures affect the occurrence, metastasis, prognosis and treatment of GBM in various aspects. For instance, researchers reported extracellular vesicles have the dual function of diagnosing GBM at an early stage and delivering Sema3A, which elevates vascular permeability, promoting invasion.^29^ The crosstalk between synaptic vesicles and the tumour microenvironment has a significant effect on the invasion and proliferation of high‐grade gliomas. For example, studies have reported that synaptic molecule neuroligin‐3 (NLGN3) could promote glioma proliferation by the PI3K‐mTOR pathway.^30^ Under the bridge of the ion channel, the interaction of GBM with reactive astrocytes was found to be closely related to the decrease in sensitivity to TMZ and the enhancement of cancer progression and aggression.^31^, ^32^

The phenomenon of neutrophil degranulation has been reported in GBM and several other human cancers.^33^, ^34^, ^35^ Furthermore, neutrophil degranulation mediated T cell functional inhibition was found to promote the growth of GBM and that immunosuppression could be blocked through arginine supplementation.^33^

In the ceRNA co‐expression network, LYZ was found to be differently expressed in several cancers.^36^, ^37^ Through participating in immunization activities by presenting antigens, LYZ regulates the tumour microenvironment and influences cancer processes.^38^ PIK3AP1 is implicated in the activation of the PI3K/AKT pathway through phosphorylation of AKT mediated by B cells and natural killer cells.^39^, ^40^ This process is also essential for cell proliferation, metabolism, cancer inhibition and oxaliplatin resistance.^41^, ^42^ These identified mRNAs are all potential biomarkers for predicting the prognosis of cancers. Previous reports have indicated that miR‐3613, miR‐373, miR‐32 and miR‐92 participate in the cancer epithelial‐mesenchymal transition (EMT) process, which is critical for cancer migratory and invasive capabilities.^43^, ^44^, ^45^, ^46^ MiR‐371 has been found to promote the proliferation and cell cycle of GBM cells, acting as a proto‐oncogene.^47^ Several lines of evidence suggest that lncRNA‐XIST induces macrophage polarization, promotes the EMT process and stimulates the progression of cancers.^48^, ^49^ LncRNA NORAD was found to be significantly associated with cell proliferation, migration and invasion, affecting apoptosis and EMT.^50^ In terms of function and structure, our enrichment analysis results are consistent with the above studies.

Previous studies have provided an experimental basis for our predicted ceRNA network. Combined with the existing research results, we speculate that the molecular mechanism of our ceRNA network might be associated with immune activities and tumour microenvironment variations. The mechanisms of the immune and tumour microenvironment in GBM are important from its initiation, and the studies of interactions among mRNAs, miRNAs and lncRNAs are currently limited. Our research has provided novel aspects to promote the study of GBM development and treatment. However, further verification experiments should be carried out in the near future to demonstrate the current conclusion.

## 5 CONCLUSIONS

In conclusion, in our ceRNA co‐expression network, the interaction of lncRNAs and miRNAs leads to the differential expression of LYZ and PIK3AP1, which then leads to a worse prognosis of GBM. We suspect the poor prognosis is mainly related to immune activities and tumour microenvironment variations. Our findings will shed light to understanding the underlying molecular mechanism of GBM and will provide new biomarkers for clinical diagnosis and treatment, and our results can be used to guide future in‐depth studies of GBM. However, its practical application value, such as sensitivity, specificity and price, has yet to be verified by laboratory studies and large‐scale clinical studies.

## CONFLICT OF INTEREST

The authors declare no competing financial interests.

## AUTHOR CONTRIBUTIONS

**Qunlong Peng:** Conceptualization (equal); Funding acquisition (equal); Project administration (equal); Resources (equal); Writing‐original draft (equal); Writing‐review & editing (equal). **Runmin Li:** Investigation (equal); Methodology (equal); Software (equal); Writing‐original draft (equal). **Ying Li:** Investigation (equal); Methodology (equal); Writing‐original draft (equal). **Xiaoqian Xu:** Validation (equal); Writing‐review & editing (equal). **Wensi Ni:** Validation (equal); Writing‐review & editing (equal). **Huiran Lin:** Validation (equal); Writing‐review & editing (equal). **Liang Ning:** Supervision (equal); Writing‐review & editing (equal).

## Data Availability

All the data were available upon request.
